# A machine learning approach for estimating Eastern Asian origins from massive screening of Y chromosomal short tandem repeats polymorphisms

**DOI:** 10.1007/s00414-024-03406-w

**Published:** 2025-01-08

**Authors:** Haeun You, Soong Deok Lee, Sohee Cho

**Affiliations:** 1 Department of Forensic Medicine, Seoul National University College of Medicine, 103 Daehak-ro, Jongno-gu, Seoul, 03080 Republic of Korea; 2 Institute of Forensic and Anthropological Science, Seoul National University Medical Research Center, 103 Daehak-ro, Jongno-gu, Seoul, 03080 Republic of Korea

**Keywords:** East Asia, Biogeographical origin, Y chromosome, Short tandem repeat, Machine learning

## Abstract

Inferring the ancestral origin of DNA evidence recovered from crime scenes is crucial in forensic investigations, especially in the absence of a direct suspect match. Ancestry informative markers (AIMs) have been widely researched and commercially developed into panels targeting multiple continental regions. However, existing forensic ancestry inference panels typically group East Asian individuals into a homogenous category without further differentiation. In this study, we screened Y chromosomal short tandem repeat (Y-STR) haplotypes from 10,154 Asian individuals to explore their genetic structure and generate an ancestry inference tool through a machine learning (ML) approach. Our research identified distinct genetic separations between East Asians and their neighboring Southwest Asians, with tendencies of northern and southern differentiation observed within East Asian populations. All machine learning models developed in this study demonstrated high accuracy, with the Asian classification model achieving an optimal performance of 82.92% and the East Asian classification model reaching 84.98% accuracy. This work not only deepens the understanding of genetic substructures within Asian populations but also showcases the potential of ML in forensic ancestry inference using extensive Y-STR data. By employing computational methods to analyze intricate genetic datasets, we can enhance the resolution of ancestry in forensic contexts involving Asian populations.

**Supplementary Information:**

The online version contains supplementary material available at 10.1007/s00414-024-03406-w.

## Introduction

East Asia is one of the most critical regions for studying human population evolution and genetic diversity. The genetic composition of contemporary East Asian populations reflects a complex history shaped by human colonization, differentiation, migrations, and admixture. Over the past few decades, substantial genetic data on East Asian populations have been accumulated [1], fostering efforts to identify and utilize informative markers that can further delineate their genetic substructure [2–4]. Earlier phylogenetic studies have revealed a north-south pattern of genetic differentiation among East Asian populations, approximately divided by the Yangtze River [5–9]. Unfortunately, most current forensic panels for ancestry inference classify East Asian populations as a single cluster, thereby limiting their efficacy in precisely determining the specific origin of unknown individuals within East Asia.

To trace the ancestry of East Asians, marked by complex genetic structures, the utilization of effective ancestry informative markers (AIMs) is essential. AIMs, which exhibit substantial allele frequency differences between geographically distinct populations, have been employed to reveal genetic differentiation among human groups across multiple continents. In particular, Y chromosomal genetic markers are favored for initial ancestry inference due to their exclusive paternal transmission and absence of recombination, which preserves distinct population-specific haplotypes [10]. Although Y-STR haplotype data have been extensively utilized to investigate the differentiation and migration patterns of East Asian populations, their application as a tool for inferring the ancestral origins of individuals within this region remains limited.

Meanwhile, to discover the genetic structure of populations, researchers have employed dimensionality reduction techniques such as principal component analysis (PCA) and have examined the proportions of genetic components of studied individuals using software like STRUCTURE and ADMIXTURE [4]. Nonetheless, these conventional approaches have limitations in accurately inferring ancestral origins for populations with closely related genetic backgrounds. Machine learning (ML) has emerged as a promising alternative methodology, enabling computational systems to autonomously learn and uncover complex attributes from high-dimensional datasets. A notable strength of ML lies in its proficiency in uncovering hidden genetic structures that may be overlooked by traditional techniques. With its robust performance in pattern recognition and classification of samples, ML offers significant potential for enhancing the efficacy of tools used for ancestry inference. Indeed, recent studies in the field of population genetics have demonstrated the remarkable utility of ML not only in analyzing genetic structure but also as advanced tool for ancestry prediction [11–14].

In this study, we assembled a comprehensive dataset of Y-STR haplotypes from Asian populations to characterize genetic differentiation between East Asian groups and their neighboring regions. We utilized various ML techniques, including Support Vector Machine (SVM), XGBoost, and Random Forest classifiers, to develop a classification system among Asian groups. To date, large-scale screening of Y-STR haplotypes utilizing ML approaches has not yet been reported in Asian populations. This study underscores the potential of using advanced computational methods to analyze complex genetic data and reveal population-specific patterns, thereby improving our understanding of genetic diversity and the resolution of ancestry inference.

## Materials and methods

### Collection of Y-STR profiles

A total of 10,154 unrelated Asian male Y-STR haplotypes were analyzed in this study. Of these, 1,008 DNA samples were either purchased from the Coriell Institute for Medical Research (Camden, NJ, USA) or directly collected as blood, blood FTA cards, or buccal swabs after obtaining written informed consent from all participants (Supplementary Table S1 [15]). The collection of samples for this study received approval from the Institutional Review Board of Seoul National University Hospital Biomedical Research Institute (IRB No. 1404-068-572). DNA extraction was carried out using the QIAamp^®^ DNA Mini Kit (QIAGEN, Hilden, Germany) for blood samples and the QIAamp^®^ DNA Investigator Kit (QIAGEN) for buccal swabs and FTA cards. Quantification of DNA was performed using the Qubit™ Flex Fluorometer (Invitrogen™, MA, USA) with the Qubit™ 1X dsDNA High Sensitivity (HS) Assay Kit (Invitrogen™) following the manufacturer’s guidelines. Multiplex amplification of Y-STR markers was executed using the PowerPlex^®^ Y23 System (Promega, Madison, WI, USA). The amplified products were separated on the 3500 Genetic Analyzer (Applied Biosystems™, Foster City, CA, USA), and the electrophoresis data were analyzed using the GeneMapper^®^ ID Software v.1.5 (Applied Biosystems™). Microvariants, multi-allele, and null alleles were verified through repeated amplification procedures. The remaining 9,146 Y-STR profiles were gathered from various literature sources (Supplementary Table S2 [16–35]).

For this study, 17 Y-STR loci from the AmpFLSTR™ Yfiler™ PCR Amplification Kit (Applied Biosystems™) were utilized, including DYS438, DYS393, DYS385a/b, DYS389I/II, DYS458, DYS437, DYS391, DYS392, DYS635, Y GATA H4, DYS19, DYS390, DYS439, DYS456, and DYS448, as these were the most abundant markers available within the entire dataset. The Asian populations were categorized into three geographical groups: Northeast Asian (NEA), covering Korea, Northern China, Japan and Mongolia; Southeast Asian (SEA), covering Southern China, Taiwan, Malaysia, the Philippines, Myanmar, and Vietnam; and Southwest Asian (SWA), covering India, Bangladesh, Nepal, Pakistan, and Sri Lanka. The Yangtze River in China served as a geographic boundary to distinguish between NEA and SEA populations, a method commonly employed in other studies.

### Population study

Allele frequencies of Y-STR loci and haplotype frequencies were determined by direct counting. Allelic diversity and haplotype diversity (HD) were calculated using Nei’s formula, (n/*n* − 1) (1 − Σp_i_^2^) [36]. The haplotype match probability (HMP) was calculated using the formula, HMP = Σp_i_^2^, where p_i_ represents the frequency of the haplotype. The discrimination capacity (DC) was estimated by assessing the proportion of unique haplotypes in each dataset. Population pairwise genetic distances (Rst values) and corresponding P-values were calculated for all given pairs among 15 subgroups (Northern Chinese, Japanese, Korean, Chinese Mongolian, Southern Chinese, Taiwanese, Malaysian, Filipino, Burmese, Vietnamese, Indian, Bangladeshi, Nepalese, Pakistani, and Sri Lankan) in each geographical group using Arlequin version 3.5.2.2 [37]. The analysis of molecular variance (AMOVA) was also performed by the same software to assess the statistical significance of genetic differentiation among groups [38]. The DYS389I PCR product represents a subset of the DYS389II amplicon, as the forward PCR primer binds to the flanking region of two repeat regions that are approximately 120 bp apart. To independently assess the variation in these regions, the number of repeats at DYS389II was calculated by subtracting the DYS389I value [39]. The DYS385 locus was excluded from the distance calculations due to the indistinguishability between its two alleles (DYS385a and DYS385b), and any samples demonstrating non-consensus alleles or null alleles at any locus were also excluded from these analyses. To illustrate the relationships between populations based on the calculated Rst values, a multidimensional scaling (MDS) plot was generated using IBM SPSS Statistics version 29, and a Neighbor-Joining (NJ) tree was constructed using MEGA software version 11.0.13 [40].

### Machine learning based ancestry inference

Data preprocessing and development of machine learning models were conducted using the Python programming language. Prior to model construction, the DYS385 locus and haplotypes containing non-consensus or null alleles were excluded, as these elements were not representative of the broader population distribution and could introduce potential bias. The entire dataset was randomly split into a training set (80%) and a test set (20%).

Supervised machine learning was performed using three algorithms from the scikit-learn library [41]: Support Vector Machine (SVM), XGBoost, and Random Forest classifiers. SVM, widely used for classification tasks, aims to identify the optimal hyperplane that maximally separates different classes based on kernel functions. To address the multi-class classification problem, one-against-all (OAA, also known as one-vs-rest) and one-against-one (OAO, also known as one-vs-one) strategies were employed (see Supplementary Fig. S1). To minimize data distortion from outliers, scaling was performed using the RobustScaler from scikit-learn. Additionally, two decision tree-based ensemble algorithms, XGBoost and Random Forest, were used. XGBoost constructs trees sequentially, with each tree trained to correct errors made by previous trees. In contrast, Random Forest builds multiple independent decision trees and combines their predictions (see Supplementary Fig. S2). Each classifier was trained on the training set with hyperparameter tuning and 5-fold cross-validation using grid search to mitigate overfitting and underfitting. Final evaluations were conducted on the test dataset. To ensure robust reproducibility, each experiment was repeated five times.

Model performance was evaluated using a confusion matrix, which categorizes predictions into four outcomes: true positive (TP), indicating the number of correctly predicted positive samples; true negative (TN), indicating the number of correctly predicted negative samples; false positive (FP), representing negative samples incorrectly classified as positive; and false negative (FN), representing positive samples incorrectly classified as negative. From these outcomes, accuracy, precision, recall, and F1 score were calculated using the ‘classification_report’ function. Given the class imbalance in the dataset, both macro and weighted averages were assessed. The macro average calculates metrics by treating all classes equally, whereas the weighted average applies weights based on the size of each class. The equations for these metrics are as follows:

1 $$\:\text{A}\text{c}\text{c}\text{u}\text{r}\text{a}\text{c}\text{y}=\:\frac{TP+TN}{TP+TN+FP+FN}$$

2 $$\:\text{P}\text{r}\text{e}\text{c}\text{i}\text{s}\text{i}\text{o}\text{n}=\:\frac{TP}{TP+FP}$$

3 $$\:\text{R}\text{e}\text{c}\text{a}\text{l}\text{l}=\:\frac{TP}{TP+FN}$$

4 $$\:\text{F}1\:\text{S}\text{c}\text{o}\text{r}\text{e}=\:\frac{TP}{TP+0.5\:(FP+FN)}$$

## Results

### Genetic diversity and population structure of Y-STRs in Asian populations

The allele frequencies and allelic diversities of 15 single-copy Y-STR loci and one multi-copy Y-STR locus are presented across each geographical group in Supplementary Table S3, Table S4, and Fig. S3. The multi-copy Y-STR locus, DYS385, demonstrated the highest level of diversity across all groups (NEA, SEA, and SWA). Among the single-copy loci, DYS458 emerged as the most diverse marker in the NEA and SEA groups, whereas DYS635 displayed the greatest variability in the SWA group. DYS391 and DYS437 exhibited lower levels of polymorphism in all groups, with DYS438 showing the least allelic diversity value particularly in the SEA group. The discrimination capacities for the NEA, SEA, and SWA populations were 0.8726, 0.8612, and 0.8506, respectively. Haplotype analysis identified a high proportion of unique haplotypes (approximately 90%) within each group, as detailed in Supplementary Table S5. A total of 30 haplotypes were common between the NEA and SEA groups, with 6 haplotypes shared between the NEA and SWA groups, and another 6 haplotypes common between the SEA and SWA populations. Notably, two haplotypes were found to be identical across all three groups.

AMOVA results revealed that the highest level of variation exists within 15 subgroups (90.30%), followed by variation among the three geographical groups (5.91%) and among subgroups within geographical groups (3.78%) (Supplementary Table S6). Pairwise genetic distances (Rst values) disclosed a close relationship between the NEA and SEA groups (Rst = 0.0365), while the distance between SEA and SWA was the greatest among the pairs (Rst = 0.1089) (Supplementary Table S7 and Table S8). The MDS plot and NJ tree (Supplementary Fig. S4 and Fig. S5), based on pairwise Rst values, demonstrated significant genetic differentiation between East Asian populations and the SWA group. Remarkably, SEA populations formed a distinct cluster, except for the Burmese population, which showed a pronounced genetic similarity with the Northern Chinese population (Rst = 0.0043).

### Machine learning-based ancestry inference among three Asian groups

**Fig. 1:**
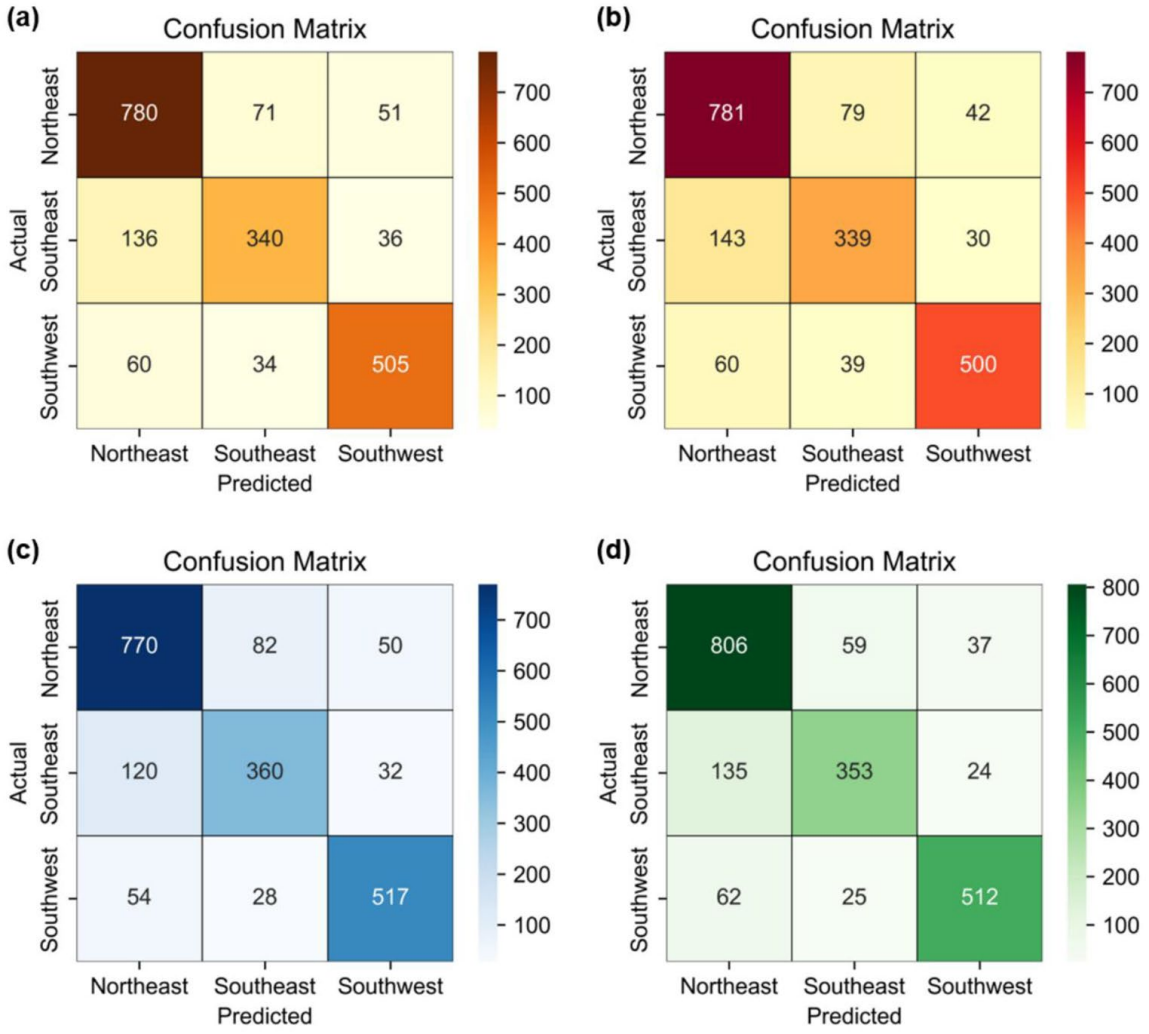
Confusion matrices of the best-performing models for Asian classification: (**a**) OAO-SVM, (**b**) OAA-SVM, (**c**) XGBoost, and (**d**) Random Forest. * Darker shades represent higher counts

To differentiate East Asian group and its neighboring populations, four machine learning models were developed and evaluated to infer the geographical origins of individuals based on their Y-STR profiles. Results from five experimental repetitions are presented in Table 1 as the mean ± standard deviation. The optimized hyperparameters for the best-performing model in each classifier are listed in Table 2, with the corresponding confusion matrices shown in Fig. 1.

**Table 1:** Evaluation metrics for classification models of Asian geographical groups

Model	Accuracy (%)	Precision	Recall	F1 score
OAO-SVM	80.73 ± 0.00	0.81 ± 0.00 (0.81 ± 0.00)	0.79 ± 0.00 (0.81 ± 0.00)	0.80 ± 0.00 (0.81 ± 0.00)
OAA-SVM	80.48 ± 0.00	0.80 ± 0.00 (0.80 ± 0.00)	0.79 ± 0.00 (0.80 ± 0.00)	0.79 ± 0.00 (0.80 ± 0.00)
XGBoost	81.82 ± 0.00	0.81 ± 0.00 (0.82 ± 0.00)	0.81 ± 0.00 (0.82 ± 0.00)	0.81 ± 0.00 (0.82 ± 0.00)
RandomForest	82.92 ± 0.08	0.83 ± 0.00 (0.83 ± 0.00)	0.81 ± 0.00 (0.83 ± 0.00)	0.82 ± 0.00 (0.83 ± 0.00)

*Precision, recall, and F1 score are presented as macro averages, with the corresponding weighted averages shown in parentheses

**Table 2:** Suitable hyperparameters for classification models of Asian geographical groups

Model	Hyperparameter
OAO-SVM	‘estimator__C’: 10, ‘estimator__gamma’: ‘scale’
OAA-SVM	‘estimator__C’: 10, ‘estimator__gamma’: ‘scale’
XGBoost	‘learning_rate’: 0.1, ‘max_depth’: 5, ‘n_estimators’: 500
RandomForest	‘max_depth’: 15, ‘min_samples_leaf’: 2, ‘min_samples_split’: 5, ‘n_estimators’: 300

**Fig. 2:**
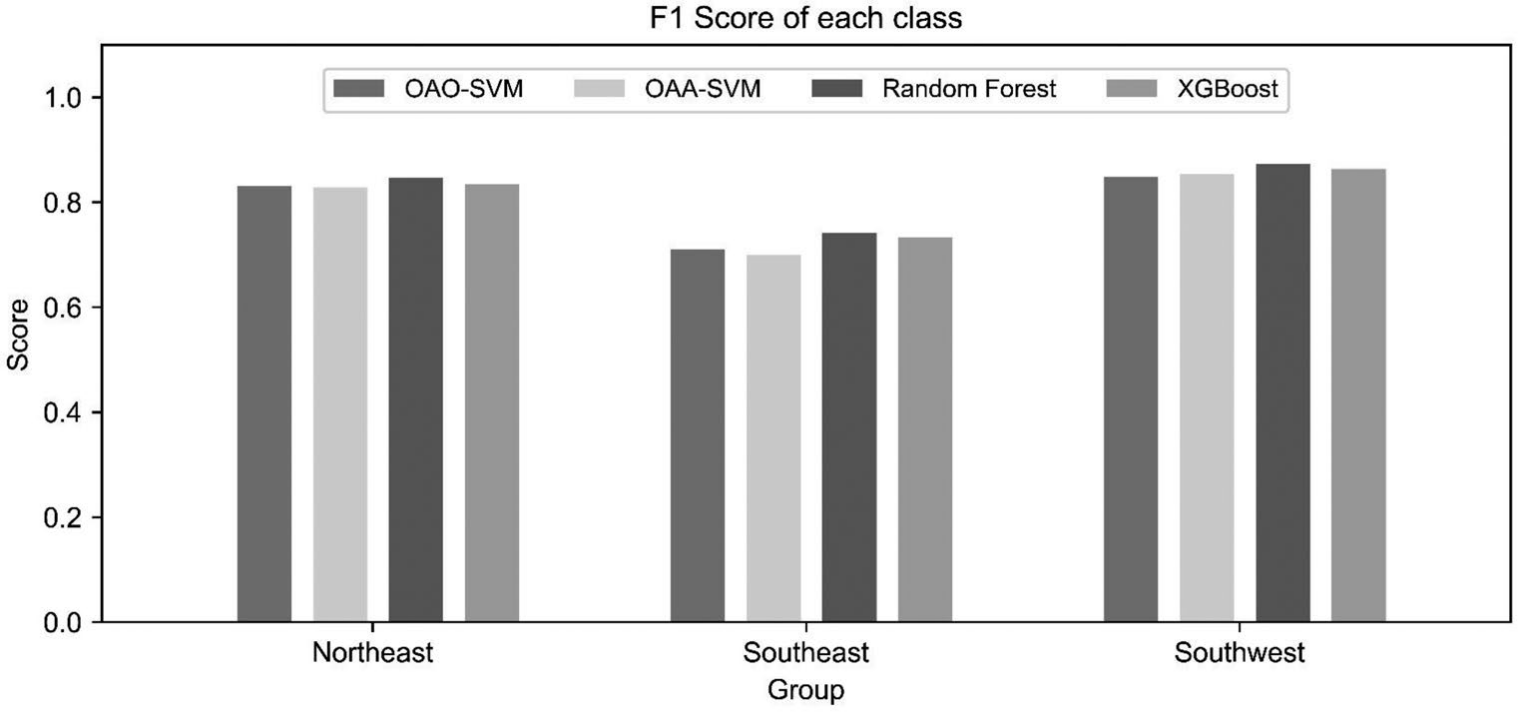
F1-score of each geographical group in Asian classification models

All models demonstrated generalization without overfitting or underfitting. Based on test accuracy, the random forest model achieved the highest performance (82.92%), followed by XGBoost (81.82%), OAO- SVM (80.73%), and OAA- SVM (80.48%). Performance across precision, recall, and F1 scores was consistent among the models, with values ranging from 0.79 to 0.83. For predictive performance across geographic groups, the SEA group consistently showed lower F1 scores than the other groups (Fig. 2). According to the confusion matrix, the NEA and SWA groups achieved prediction accuracies above 80% in all models, whereas accuracy for the SEA group ranged between 66.21% and 70.31% (Fig. 1).

**Fig. 3:**
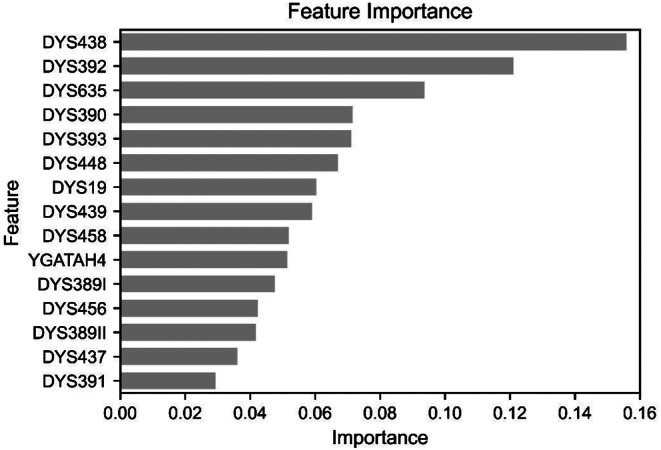
Feature importance of the best-performing random forest models for Asian classification

To further analyze the factors contributing to the high performance of the random forest model, feature importance was extracted and presented in Fig. 3. DYS438, DYS392, and DYS635 exhibited particularly high importance in the model, while DYS391 and DYS437 showed the lowest importance.

### Machine learning-based ancestry inference between two east Asian groups

**Fig. 4:**
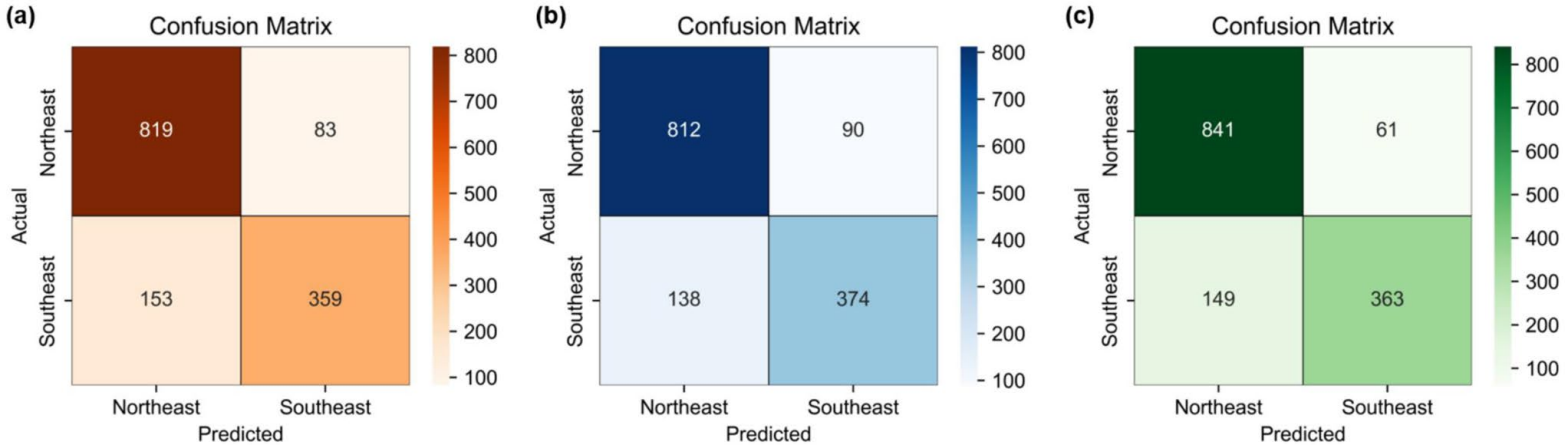
Confusion matrices of the best-performing models for East Asian classification: (**a**) SVM, (**b**) XGBoost, and (**c**) Random Forest. * Darker shades represent higher counts

Refined models were developed and evaluated specifically for distinguishing between two East Asian groups. Results from five experimental repetitions are presented in Table 3 as the mean ± standard deviation. The optimized hyperparameters for the best-performing model in each classifier are listed in Table 4 with the corresponding confusion matrices shown in Fig. 4.

**Table 3:** Evaluation metrics for classification models of east Asian geographical groups

Model	Accuracy (%)	Precision	Recall	F1 score
SVM	83.31 ± 0.00	0.83 ± 0.00 (0.83 ± 0.00)	0.80 ± 0.00 (0.83 ± 0.00)	0.81 ± 0.00 (0.83 ± 0.00)
XGBoost	83.88 ± 0.00	0.83 ± 0.00 (0.84 ± 0.00)	0.82 ± 0.00 (0.84 ± 0.00)	0.82 ± 0.00 (0.84 ± 0.00)
RandomForest	84.98 ± 0.16	0.85 ± 0.00 (0.85 ± 0.00)	0.82 ± 0.00 (0.85 ± 0.00)	0.83 ± 0.00 (0.85 ± 0.00)

*Precision, recall, and F1 score are presented as macro averages, with the corresponding weighted averages shown in parentheses

**Table 4:** Suitable hyperparameters for classification models of east Asian geographical groups

Model	Hyperparameter
SVM	‘C’: 10, ‘gamma’: 0.1
XGBoost	‘learning_rate’: 0.1, ‘max_depth’: 5, ‘n_estimators’: 500
RandomForest	‘max_depth’: 15, ‘min_samples_leaf’: 2, ‘min_samples_split’: 5, ‘n_estimators’: 200

**Fig. 5:**
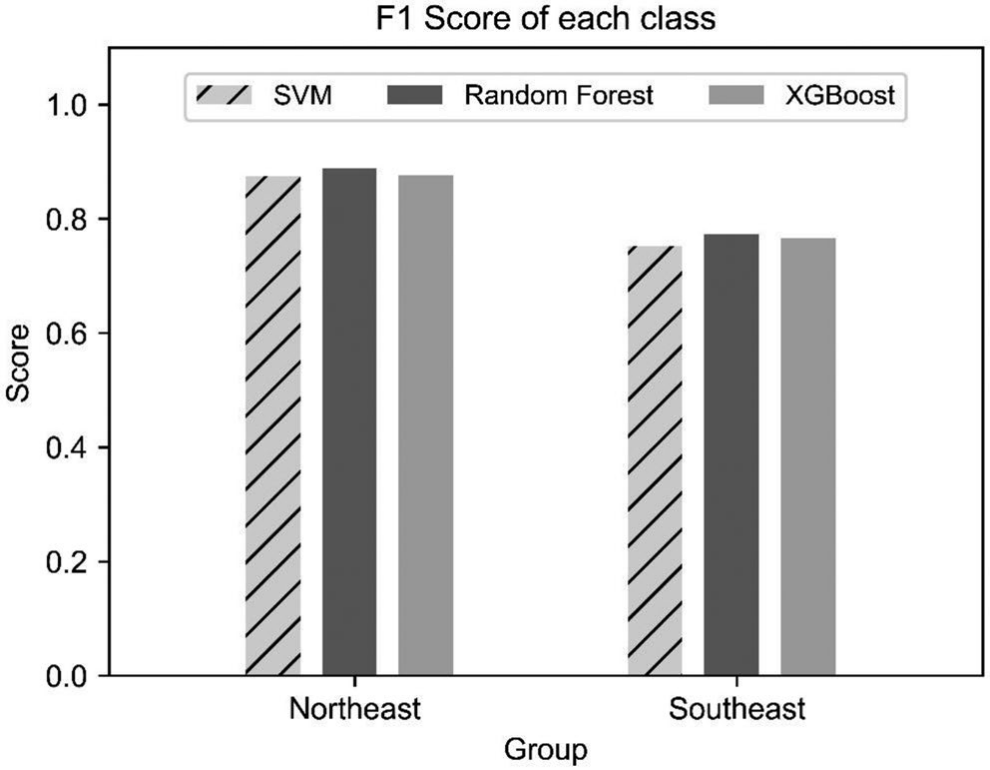
F1-score of each geographical group in East Asian classification models

All models demonstrated generalization without overfitting or underfitting. Based on test accuracy, the random forest achieved the highest performance (84.98%), followed by XGBoost (83.88%), and SVM (83.31%). Performance across precision, recall, and F1 scores was consistent among the models, with values ranging from 0.80 to 0.85. In terms of predictive performance across geographic groups, the SEA group consistently showed lower F1 scores than the NEA group (Fig. 5). According to the confusion matrix, the NEA group was predicted with 90% accuracy, whereas the SEA group achieved only 70% accuracy, with approximately 365 of 512 test samples correctly classified (Fig. 4).

**Fig. 6:**
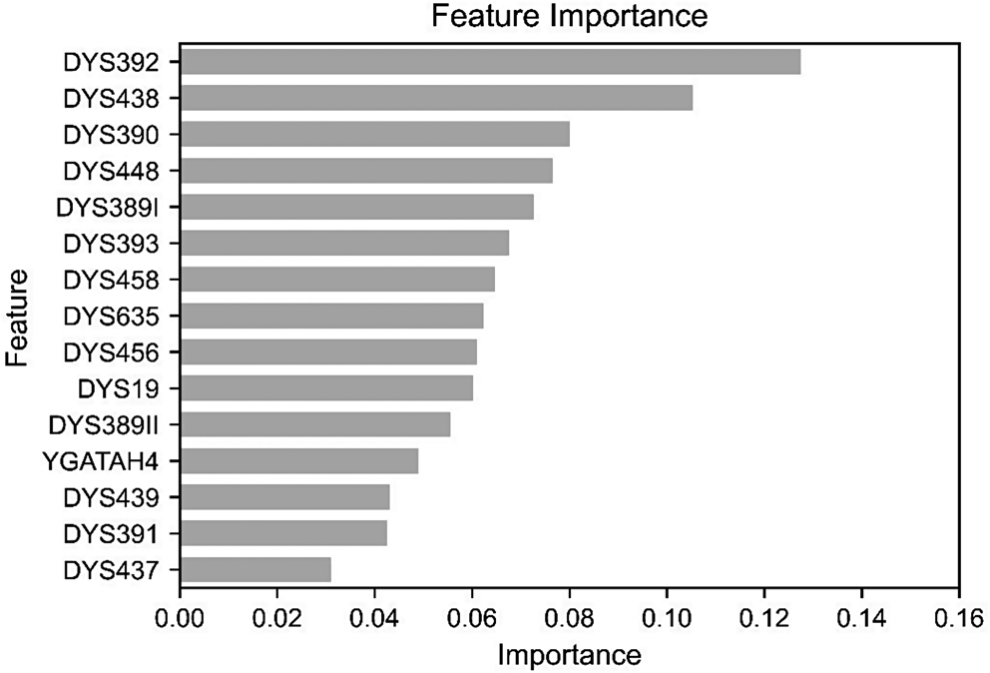
Feature importance of the best-performing random forest models for East Asian classification

To further analyze the factors contributing to the high performance of the random forest model, feature importance was extracted and presented in Fig. 6. DYS392, DYS438, and DYS390 exhibited particularly high importance in the model, while DYS391 and DYS437 showed the lowest importance.

## Discussion

Asia, home to 60% of the world’s population is characterized by diverse ethnic groups with complex genetic structures. Population genetic studies of Asian populations have advanced significantly since the initial Human Genome Organization (HUGO) Pan-Asian SNP Consortium [42] and now include large-scale efforts such as the GenomeAsia 100 K project [43]. The accumulation of genetic data and advancements in experimental techniques have made it possible to study both ancient and modern human genomes with high precision. This progress has enabled researchers to investigate the genetic history of Asian populations over the past 45,000 years, revealing differentiation into three primary ancestral lineages: Ancient Ancestral South Indian (AASI), Australasian (AA), and East and Southeast Asian (ESEA) [44]. Consistent with prior studies, the genetic structure analysis conducted in this study demonstrates distinct genetic differentiation between Southwest Asian and East Asian groups. Furthermore, our extensive Y-STR analysis supports the notion that East Asian populations can be genetically subdivided into northern and southern clusters along the Yangtze River. Within the East Asian cluster, subgroup analysis revealed a close genetic relationship between Korean and Japanese populations, which likely originates from the geographical connectivity between the Korean Peninsula and Japanese archipelago before their separation due to rising sea levels [45]. Meanwhile, the Northern Chinese subgroup exhibits high genetic affinity with Southeast Asian groups, particularly the Myanmar population. This pattern may be attributed to historical migration patterns, such as the southward movement of Tibeto-Burman populations from northwestern China approximatel 2,600 years ago [46]. However, when inferring specific ethnic relationships, it is important to note that historical factors, including social structures and marriage patterns, may impact interpretation. Furthermore, results can vary significantly depending on the reference datasets used.

To gain a deeper understanding of the complex genetic structure of continental regions, researchers have increasingly employed machine learning approaches in biogeographical ancestry studies. However, for East Asians, who exhibit intricate migration patterns, research remains limited. Sun et al. [4] developed a method to predict the geographic origins of East Asian populations using insertion-deletion polymorphisms(InDels) and machine learning algorithms. Their study demonstrated the outstanding performance of ML methods for forensic ancestry inference, achieving an accuracy of approximately 80% using multi-indel markers, the best performing model achieving 87%. It is important to consider that the cohort used in their study differed from the subjects in our research. The authors included Malaysian, Laotian, and Cambodian samples in the Southeast Asian group but excluded ethnic groups adjacent to Northeast Asia, such as the Southern Chinese populations. The individual genotypic differences in their training data may have contributed to more effective learning and classification compared to the groups we studied. In contrast, given the extensive diversity among Asian populations, our model aimed to predict geographic origin by incorporating data from broad regions. To improve classification of East Asian populations, this study developed a model to identify East Asians among broader Asian groups and further categorize them into Northeast and Southeast clusters. Machine learning approaches, with their ability to efficiently handle large-scale datasets, were particularly well-suited for this task and offer flexibility for future data integration.

In this study, we selected three classification algorithms. SVM was chosen for its effectiveness in handling high-dimensional data, while decision tree models were utilized for their ability to provide interpretable predictions through tree-based structures. Our findings indicated that tree-based models using raw data outperformed SVM models with transformed data. Although it is challenging to pinpoint the exact reasons for this performance difference due to SVM’s black-box nature, the superior performance of tree-based models may be attributed to their capacity for capturing potential interactions between markers, or alternatively, the scaling applied to SVM might have diluted the relationships between STR values. In forensic DNA analysis, the transparency of the result-generation process is crucial [47]. The combination of superior performance and the white-box nature of tree-based models, which allows for visual examination of the model construction process, enhances transparency and reliability in forensic applications.

Four key metrics were investigated to evaluate model performance. Accuracy represents the proportion of correctly predicted samples in the entire sample set. Precision indicates the ratio of true positives among all samples predicted as positive, making it particularly useful when minimizing false positives is essential. Recall represents the ratio of true positives among all actual positive samples, which is crucial when avoiding false negatives. The F1 score, calculated as the harmonic mean of precision and recall, is widely used in evaluating imbalanced datasets in multi-class classification [48]. Given the slight class imbalance in this study, we focused on both accuracy and F1 score. All machine learning models demonstrated the capability to classify Asian geographical groups with accuracy exceeding 80% and achieved F1 scores of approximately 0.80 overall (Tables 1 and 3). High F1 scores generally indicate balanced performance, suggesting that the models achieved both high precision and high recall. In this study, models maintained stable performance with F1 scores consistently around 0.80. However, in subgroup-specific analyses, the SEA group showed relatively lower F1 scores (Figs. 2 and 5). This performance difference likely results from the smaller training dataset available for this group compared to others. Future research should incorporate additional data for Southeast Asian populations.

Compared to classical statistical analysis, machine learning approaches have the advantage of analyzing patterns among all genetic markers in the training dataset, selecting those with the highest predictive power. Feature importance indicates the contribution of each feature to tree-based decision-making, measured on a scale from 0 to 1. Values closer to 1 indicate greater predictive accuracy, and the sum of all feature importance values equals 1 [48]. Markers with low mutation rates are generally suitable for ancestry inference, as they better reflect human history. While stable markers were expected to have high importance in machine learning models, our exploratory analysis did not reveal statistically significant relationships between feature importance and genetic characteristics such as mutation rates or genetic diversity. This suggests that the model’s feature selection may be influenced by complex patterns beyond basic genetic attributes, although further investigation with an expanded set of markers would be necessary to confirm this observation. Future research could benefit from employing a broader array of STR markers, selecting only those that are most informative, potentially leading to the development of more effective models. However, it is essential to recognize that feature importance rankings are specific to the model used, necessitating the careful selection of markers that align with the particular model employed.

Meanwhile, the Y chromosome serves as a potent genetic marker due to its capacity to provide male-specific genetic information, which is particularly valuable in forensic cases involving sexual assaults where distinguishing male DNA from a female background is crucial [49]. This study selected Y-chromosomal markers to utilize these forensic advantages. Y-STR markers are particularly significant, as they are widely represented in public databases, owing to their integration into standard genetic testing procedures. Previous research has shown that Y-chromosomal single nucleotide polymorphisms (Y-SNPs) possess strong geographical specificity, enabling direct assessment of admixture patterns among diverse populations [50, 51]. Additionally, Y-chromosomal insertion-deletion polymorphisms (Y-InDels) are useful tools for tracing biogeographical origins [52]. The strategic combination of these Y-chromosomal markers is expected to enhance the resolution of population genetic analyses [53]. It is important to note, however, that DNA variants on the Y chromosome represent a single paternal lineage and may not fully capture an individual’s overall ancestry, particularly when distant maternal lineages with unusual ancestry are inherited [10]. This limitation can be addressed by incorporating autosomal genetic markers. Future studies that include diverse marker types may provide a more comprehensive understanding of population genetic history.

## Conclusion

In this study, we conducted a comprehensive analysis of Y-STR haplotypes across a broad dataset of Asian populations to explore their genetic diversity and differentiation. Our analysis highlighted distinct genetic profiles between East Asian and Southwest Asian groups, underscored by a pronounced north-south pattern of genetic differentiation within East Asia. The deployment of machine learning techniques showed high accuracy in classifying individuals into their respective Asian groups based on Y-STR haplotypes. These findings offer valuable insights into the ancestry of populations in East Asia and adjacent regions, presenting machine learning models based on Y-STR markers as promising predictive tools. Future research, incorporating a broader array of genetic markers and enhanced computational techniques, could further improve the resolution of ancestry.

## Electronic supplementary material

Below is the link to the electronic supplementary material.

Supplementary Material 1

Supplementary Material 2

## References

[1] Pan ZQ, Xu SH (2020) Population genomics of east Asian ethnic groups. Hereditas 157:49. 10.1186/s41065-020-00162-w 33292737 10.1186/s41065-020-00162-w PMC7724877

[2] Li CX, Pakstis AJ, Jiang L, Wei YL, Sun QF, Wu H, Bulbul O, Wang P, Kang LL, Kidd JR, Kidd KK (2016) A panel of 74 AISNPs: Improved ancestry inference within Eastern Asia. Forensic Sci International: Genet 23:101–110. 10.1016/j.fsigen.2016.04.002 10.1016/j.fsigen.2016.04.002 27077960

[3] Cao Y, Zhu Q, Huang Y, Li X, Wei Y, Wang H, Zhang J (2022) An efficient ancestry informative SNPs panel for further discriminating east Asian populations. Electrophoresis 43:1774–1783. 10.1002/elps.202100349 35749689 10.1002/elps.202100349

[4] Sun K, Yao Y, Yun L, Zhang C, Xie J, Qian X, Tang Q, Sun L (2022) Application of machine learning for ancestry inference using multi-InDel markers. Forensic Sci International: Genet 59:102702. 10.1016/j.fsigen.2022.102702 10.1016/j.fsigen.2022.102702 35378426

[5] Du R, Xiao C, Cavalli-Sforza L (1997) Genetic distances between Chinese populations calculated on gene frequencies of 38 loci. Sci China Ser C: Life Sci 40:613–621. 10.1007/BF02882691 18726285 10.1007/BF02882691

[6] Su B, Xiao J, Underhill P, Deka R, Zhang W, Akey J, Huang W, Shen D, Lu D, Luo J (1999) Y-Chromosome evidence for a northward migration of modern humans into Eastern Asia during the last ice age. Am J Hum Genet 65:1718–1724. 10.1086/302680 10577926 10.1086/302680 PMC1288383

[7] Zhang F, Su B, Zhang Y-p, Jin L (2007) Genetic studies of human diversity in East Asia. Philosophical Trans Royal Soc B: Biol Sci 362:987–996. 10.1098/rstb.2007.2028 10.1098/rstb.2007.2028 PMC2435565 17317646

[8] Zhong H, Shi H, Qi X-B, Duan Z-Y, Tan P-P, Jin L, Su B, Ma RZ (2011) Extended Y chromosome investigation suggests postglacial migrations of modern humans into East Asia via the northern route. Mol Biol Evol 28:717–727. 10.1093/molbev/msq247 20837606 10.1093/molbev/msq247

[9] Di D, Sanchez-Mazas A (2011) Challenging views on the Peopling history of East Asia: the Story according to HLA markers. Am J Phys Anthropol 145:81–96. 10.1002/ajpa.21470 21484761 10.1002/ajpa.21470

[10] Phillips C (2015) Forensic genetic analysis of bio-geographical ancestry. Forensic Sci International: Genet 18:49–65. 10.1016/j.fsigen.2015.05.012 10.1016/j.fsigen.2015.05.012 26013312

[11] Kloska A, Giełczyk A, Grzybowski T, Płoski R, Kloska SM, Marciniak T, Pałczyński K, Rogalla-Ładniak U, Malyarchuk BA, Derenko MV (2023) A machine-learning-based Approach to Prediction of Biogeographic Ancestry within Europe. Int J Mol Sci 24:15095. 10.3390/ijms242015095 37894775 10.3390/ijms242015095 PMC10606184

[12] Alladio E, Poggiali B, Cosenza G, Pilli E (2022) Multivariate statistical approach and machine learning for the evaluation of biogeographical ancestry inference in the forensic field. Sci Rep 12:8974. 10.1038/s41598-022-12903-0 35643723 10.1038/s41598-022-12903-0 PMC9148302

[13] Qu Y, Tran D, Ma WL (2019) Deep Learning Approach to Biogeographical Ancestry Inference. Procedia Comput Sci 159:552–561. 10.1016/j.procs.2019.09.210

[14] Jin XY, Liu YL, Zhang YY, Li YL, Chen CL, Wang HD (2021) Autosomal deletion/insertion polymorphisms for global stratification analyses and ancestry origin inferences of different continental populations by machine learning methods. Electrophoresis 42:1473–1479. 10.1002/elps.202100044 33948979 10.1002/elps.202100044

[15] Lee JH, Cho S, Kim MY, Shin DH, Rakha A, Shinde V, Lee SD (2018) Genetic resolution of applied biosystems (TM) precision ID ancestry panel for seven Asian populations. Leg Med 34:41–47. 10.1016/j.legalmed.2018.08.007 10.1016/j.legalmed.2018.08.007 30153533

[16] Bai R, Liu Y, Zhang J, Shi M, Dong H, Ma S, Bai RF, Shi M (2016) Analysis of 27 Y-chromosomal STR haplotypes in a Han population of Henan province, Central China. Int J Legal Med 130:1191–1194. 10.1007/s00414-016-1326-3 26932866 10.1007/s00414-016-1326-3

[17] Li XB, Zhang JS, Li LL, Zha L, Shi MS, Ding MX (2020) Genetic polymorphism of 24 Y-STR loci in Altay Hui and Kazakh populations from northwest China. Leg Med 47:101760. 10.1016/j.legalmed.2020.101760 10.1016/j.legalmed.2020.101760 32739877

[18] Hara M, Kido A, Takada A, Adachi N, Saito K (2007) Genetic data for 16 Y-chromosomal STR loci in Japanese. Leg Med 9:161–170. 10.1016/j.legalmed.2006.11.002 10.1016/j.legalmed.2006.11.002 17197226

[19] Watahiki H, Fujii K, Fukagawa T, Mita Y, Kitayama T, Mizuno N (2019) Polymorphisms and microvariant sequences in the Japanese population for 25 Y-STR markers and their relationships to Y-chromosome haplogroups. Forensic Sci International: Genet 41:e1–e7. 10.1016/j.fsigen.2019.03.004 10.1016/j.fsigen.2019.03.004 30948258

[20] Jeong KS, Shin H, Lee SJ, Kim HS, Kim JY, Han MS, Lee YH, Park KW, Chun BW (2018) Genetic characteristics of Y-chromosome short tandem repeat haplotypes from cigarette butt samples presumed to be smoked by North Korean men. Genes Genomics 40:819–824. 10.1007/s13258-018-0701-5 30047114 10.1007/s13258-018-0701-5

[21] Jung JY, Park JH, Oh YL, Kwon HS, Park HC, Park KH, Kim EH, Lee DS, Lim SK (2016) Forensic genetic study of 29 Y-STRs in Korean population. Leg Med 23:17–20. 10.1016/j.legalmed.2016.09.001 10.1016/j.legalmed.2016.09.001 27890097

[22] Wang YQ, Li SY, Dang Z, Kong X, Zhang YJ, Ma L, Wang D, Zhang H, Li CZ, Cui W (2019) Genetic diversity and haplotype structure of 27 Y-STR loci in a yanbian Korean population from Jilin Province, Northeast China. Leg Med 36:110–112. 10.1016/j.legalmed.2018.11.010 10.1016/j.legalmed.2018.11.010 30502537

[23] Gao TZ, Yun LB, Gao S, Gu Y, He W, Luo HB, Hou YP (2016) Population genetics of 23 Y-STR loci in the Mongolian minority population in Inner Mongolia of China. Int J Legal Med 130:1509–1511. 10.1007/s00414-016-1433-1 27515831 10.1007/s00414-016-1433-1

[24] Fu XL, Fu Y, Liu Y, Guo JJ, Liu YF, Guo YD, Yan J, Cai JF, Liu JS, Zha L (2016) Genetic polymorphisms of 26 Y-STR loci in the Mongolian minority from Horqin district, China. Int J Legal Med 130:941–946. 10.1007/s00414-016-1387-3 27188626 10.1007/s00414-016-1387-3

[25] Jiang W, Gong Z, Rong H, Guan H, Zhang T, Zhao Y, Fu X, Zha L, Jin C, Ding Y (2017) Population genetics of 26 Y-STR loci for the Han ethnic in Hunan province, China. Int J Legal Med 131:115–117. 10.1007/s00414-016-1411-7 27448570 10.1007/s00414-016-1411-7

[26] Luo Y, Wu Y, Qian E, Wang Q, Wang Q, Zhang H, Wang X, Zhang H, Yang M, Ji J (2019) Population genetic analysis of 36 Y-chromosomal STRs yields comprehensive insights into the forensic features and phylogenetic relationship of Chinese Tai-Kadai-Speaking Bouyei. PLoS ONE 14:e0224601. 10.1371/journal.pone.0224601 31703068 10.1371/journal.pone.0224601 PMC6839857

[27] Hwa HL, Tseng LH, Ko TM, Chang YY, Yin HY, Su YN, Lee JCI (2010) Seventeen Y-chromosomal short tandem repeat haplotypes in seven groups of population living in Taiwan. Int J Legal Med 124:295–300. 10.1007/s00414-010-0425-9 20179958 10.1007/s00414-010-0425-9

[28] Chang YM, Swaran Y, Phoon YK, Sothirasan K, Sim HT, Lim KB, Kuehn D (2009) Haplotype diversity of 17 Y-chromosomal STRs in three native Sarawak populations (Iban, Bidayuh and Melanau) in East Malaysia. Forensic Sci International: Genet 3:e77–e80. 10.1016/j.fsigen.2008.07.007 10.1016/j.fsigen.2008.07.007 19414156

[29] Hakim HM, Khan HO, Ismail SA, Lalung J, Kofi AE, Nelson BR, Abdullah MT, Chambers GK, Edinur HA (2020) Population data for 23 Y chromosome STR loci using the Powerplex^®^ Y23 STR kit for the Kedayan population in Malaysia. Int J Legal Med 134:1335–1337. 10.1007/s00414-019-02237-4 31897667 10.1007/s00414-019-02237-4

[30] Nazir M, Alhaddad H, Alenizi M, Alenizi H, Taqi Z, Sanqoor S, Alrazouqi A, Hassan A, Alfalasi R, Gaur S, Al Jaber J, Ziab J, Al-Harbi E, Moura-Neto RS, Budowle B (2016) A genetic overview of 23Y-STR markers in UAE population. Forensic Sci International: Genet 23:150–152. 10.1016/j.fsigen.2016.04.009 10.1016/j.fsigen.2016.04.009 27124011

[31] Ghosh T, Kalpana D, Mukerjee S, Mukherjee M, Sharma AK, Nath S, Rathod VR, Thakar MK, Jha GN (2011) Genetic diversity of 17 Y-short tandem repeats in Indian population. Forensic Sci International: Genet 5:363–367. 10.1016/j.fsigen.2010.12.007 10.1016/j.fsigen.2010.12.007 21277272

[32] Yadav B, Raina A, Das Dogra T (2011) Haplotype diversity of 17 Y-chromosomal STRs in Saraswat Brahmin Community of North India. Forensic Sci International: Genet 5:e63–e70. 10.1016/j.fsigen.2010.09.012 10.1016/j.fsigen.2010.09.012 20971692

[33] Mohapatra BK, Chauhan K, Shrivastava P, Sharma A, Dagar S, Kaitholia K (2019) Haplotype data for 17 Y-STR loci in the population of Himachal Pradesh, India. Int J Legal Med 133:1401–1402. 10.1007/s00414-019-02080-7 31154495 10.1007/s00414-019-02080-7

[34] Hasan M, Sufian A, Momtaz P, Mazumder AK, Khondaker JA, Bhattacharjee S, Chakma K, Akhteruzzaman S (2018) Phylogenetic analysis and forensic evaluation among Rakhine, Marma, Hajong, and Manipuri tribes from four culturally defined regions of Bangladesh using 17 Y-chromosomal STRs. Int J Legal Med 132:1641–1644. 10.1007/s00414-018-1915-4 30143861 10.1007/s00414-018-1915-4

[35] Hasan M, Momtaz P, Hosen I, Das SA, Akhteruzzaman S (2015) Population genetics of 17 Y-chromosomal STRs loci in Garo and Santal tribal populations in Bangladesh. Int J Legal Med 129:251–252. 10.1007/s00414-014-0981-5 24577712 10.1007/s00414-014-0981-5

[36] Nei M (1987) Molecular evolutionary genetics. Columbia university

[37] Excoffier L, Laval G, Schneider S (2005) Arlequin (version 3.0): an integrated software package for population genetics data analysis. Evolutionary Bioinf 1:117693430500100003. 10.1177/117693430500100003 PMC2658868 19325852

[38] Kayser M, Brauer S, Schädlich H, Prinz M, Batzer MA, Zimmerman PA, Boatin BA, Stoneking M (2003) Y chromosome STR haplotypes and the genetic structure of US populations of African, European, and hispanic ancestry. Genome Res 13:624–634. 10.1101/gr.463003 12671003 10.1101/gr.463003 PMC430174

[39] Butler JM (2011) Advanced topics in forensic DNA typing: methodology. Academic

[40] Tamura K, Stecher G, Kumar S (2021) MEGA11: molecular evolutionary genetics analysis version 11. Mol Biol Evol 38:3022–3027. 10.1093/molbev/msab120 33892491 10.1093/molbev/msab120 PMC8233496

[41] Pedregosa F, Varoquaux G, Gramfort A, Michel V, Thirion B, Grisel O, Blondel M, Prettenhofer P, Weiss R, Dubourg V (2011) Scikit-learn: machine learning in Python. J Mach Learn Res 12:2825–2830

[42] Ngamphiw C, Assawamakin A, Xu S, Shaw PJ, Yang JO, Ghang H, Bhak J, Liu E, Tongsima S, Consortium HP-AS (2011) PanSNPdb: the pan-asian SNP genotyping database. PLoS ONE 6:e21451. 10.1371/journal.pone.0021451 21731755 10.1371/journal.pone.0021451 PMC3121791

[43] GenomeAsia100K Consortium (2019) The GenomeAsia 100K Project enables genetic discoveries across Asia. Nature 576:106–111. 10.1038/s41586-019-1793-z 31802016 10.1038/s41586-019-1793-z PMC7054211

[44] Yang MA (2022) A genetic history of migration, diversification, and admixture in Asia. Hum Popul Genet Genomics 2. 10.47248/hpgg2202010001

[45] Horai S, Murayama K, Hayasaka K, Matsubayashi S, Hattori Y, Fucharoen G, Harihara S, Park KS, Omoto K, Pan I-H (1996) mtDNA polymorphism in east Asian populations, with special reference to the peopling of Japan. Am J Hum Genet 59:579 8751859 PMC1914908

[46] Wen B, Xie X, Gao S, Li H, Shi H, Song X, Qian T, Xiao C, Jin J, Su B (2004) Analyses of genetic structure of Tibeto-Burman populations reveals sex-biased admixture in southern Tibeto-burmans. Am J Hum Genet 74:856–865. 10.1086/386292 15042512 10.1086/386292 PMC1181980

[47] Barash M, McNevin D, Fedorenko V, Giverts P (2023) Machine learning applications in forensic DNA profiling: a critical review. Forensic Sci International: Genetics: 102994. 10.1016/j.fsigen.2023.102994 10.1016/j.fsigen.2023.102994 38086200

[48] Müller AC, Guido S (2016) Introduction to machine learning with Python: a guide for data scientists. O’Reilly Media, Inc

[49] Roewer L (2009) Y chromosome STR typing in crime casework. Forensic science. Med Pathol 5:77–84. 10.1007/s12024-009-9089-5 10.1007/s12024-009-9089-5 19455440

[50] Hammer MF, Chamberlain VF, Kearney VF, Stover D, Zhang G, Karafet T, Walsh B, Redd AJ (2006) Population structure of Y chromosome SNP haplogroups in the United States and forensic implications for constructing Y chromosome STR databases. Forensic Sci Int 164:45–55. 10.1016/j.forsciint.2005.11.013 16337103 10.1016/j.forsciint.2005.11.013

[51] Song M, Wang Z, Zhang Y, Zhao C, Lang M, Xie M, Qian X, Wang M, Hou Y (2019) Forensic characteristics and phylogenetic analysis of both Y-STR and Y-SNP in the Li and Han ethnic groups from Hainan Island of China. Forensic Sci International: Genet 39:e14–e20. 10.1016/j.fsigen.2018.11.016 10.1016/j.fsigen.2018.11.016 30522950

[52] Wang Z, Song M, Lyu Q, Ying J, Wu Q, Song F, Wang X, Jiang L, Zhou Y, Sun C (2024) Development and evaluation of a panel of newly screened Y chromosome InDels for inferring paternal ancestry information in Southwest China. Int J Legal Med 1–13. 10.1007/s00414-024-03344-7 10.1007/s00414-024-03344-7 39377930

[53] Zhou Z, Li Z, Yao Y, Qian J, Ji Q, Shao C, Xie J (2023) Validation of phylogenetic informative Y-InDels in Y-chromosomal haplogroup O-M175. Front Genet 14:1182028. 10.3389/fgene.2023.1182028 37205119 10.3389/fgene.2023.1182028 PMC10185902

