# Supplementary material for: A machine learning approach for estimating Eastern Asian origins from massive screening of Y chromosomal short tandem repeats polymorphisms
Source: Int J Legal Med. 2025 Jan 8;139(2):531–40. doi: 10.1007/s00414-024-03406-w (PMC11850560; doi:10.1007/s00414-024-03406-w)
Supplement: Supplementary file 2 — Supplementary Material 2 [file 414_2024_3406_MOESM2_ESM.docx]

**A machine learning approach for estimating Eastern Asian origins from massive screening of Y chromosomal short tandem repeats polymorphisms**

International Journal of Legal Medicine

Haeun You^1^, Soong Deok Lee^1,2^, Sohee Cho^2^

^1^Department of Forensic Medicine, Seoul National University College of Medicine, Seoul, Republic of Korea.

^2^Institute of Forensic and Anthropological Science, Seoul National University Medical Research Center, Seoul, Republic of Korea.

Corresponding author: Sohee Cho ([ssoya311@snu.ac.kr](mailto:sdlee@snu.ac.kr))

**Supplementary Fig. S1 Two strategies for multi-class classification in support vector machine**

**Supplementary Fig. S2 Two types of ensemble decision tree models**

**Supplementary Fig. S3 Allelic diversity of 17 Y-STR markers across three geographical groups**

**Supplementary Fig. S4 Multidimensional scaling (MDS) plot illustrating the genetic relationships among 15 subgroups**

**Supplementary Fig. S5 Neighbor-joining (NJ) trees illustrating the genetic relationships among Asian populations**

**Supplementary Fig. S1 Two strategies for multi-class classification in Support Vector Machine**

(a) the One-against-All approach involves training a separate classifier for each class, and (b) the One-against-One approach involves training a separate classifier for each pair of classes

**
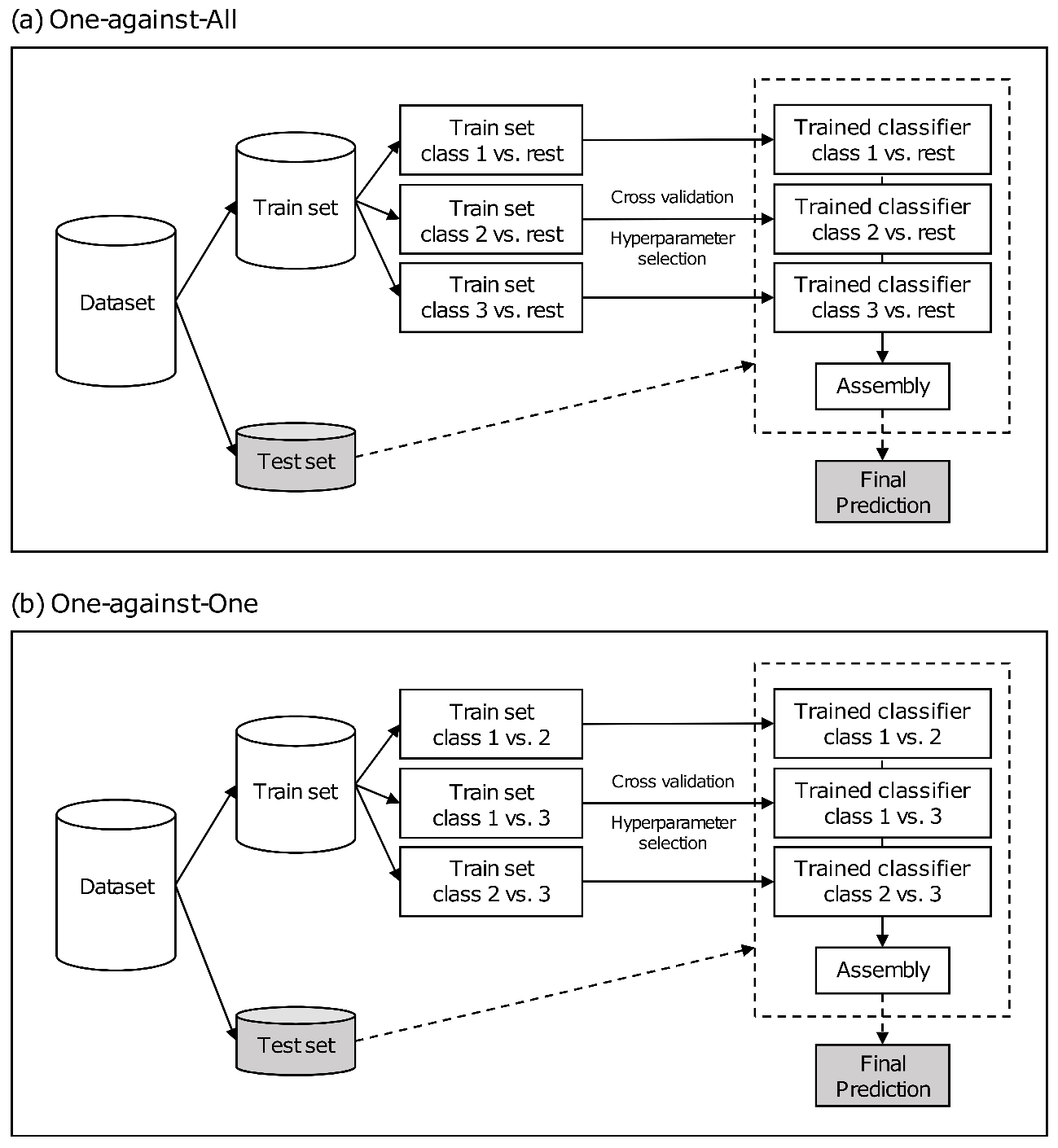
**

**Supplementary Fig. S2 Two types of ensemble decision tree models**

(a) XGBoost, a boosting-based method that progressively integrates weak classifiers to minimize training errors, and (b) Random Forest, a bagging-based method that simultaneously trains multiple decision trees using randomly selected features

**
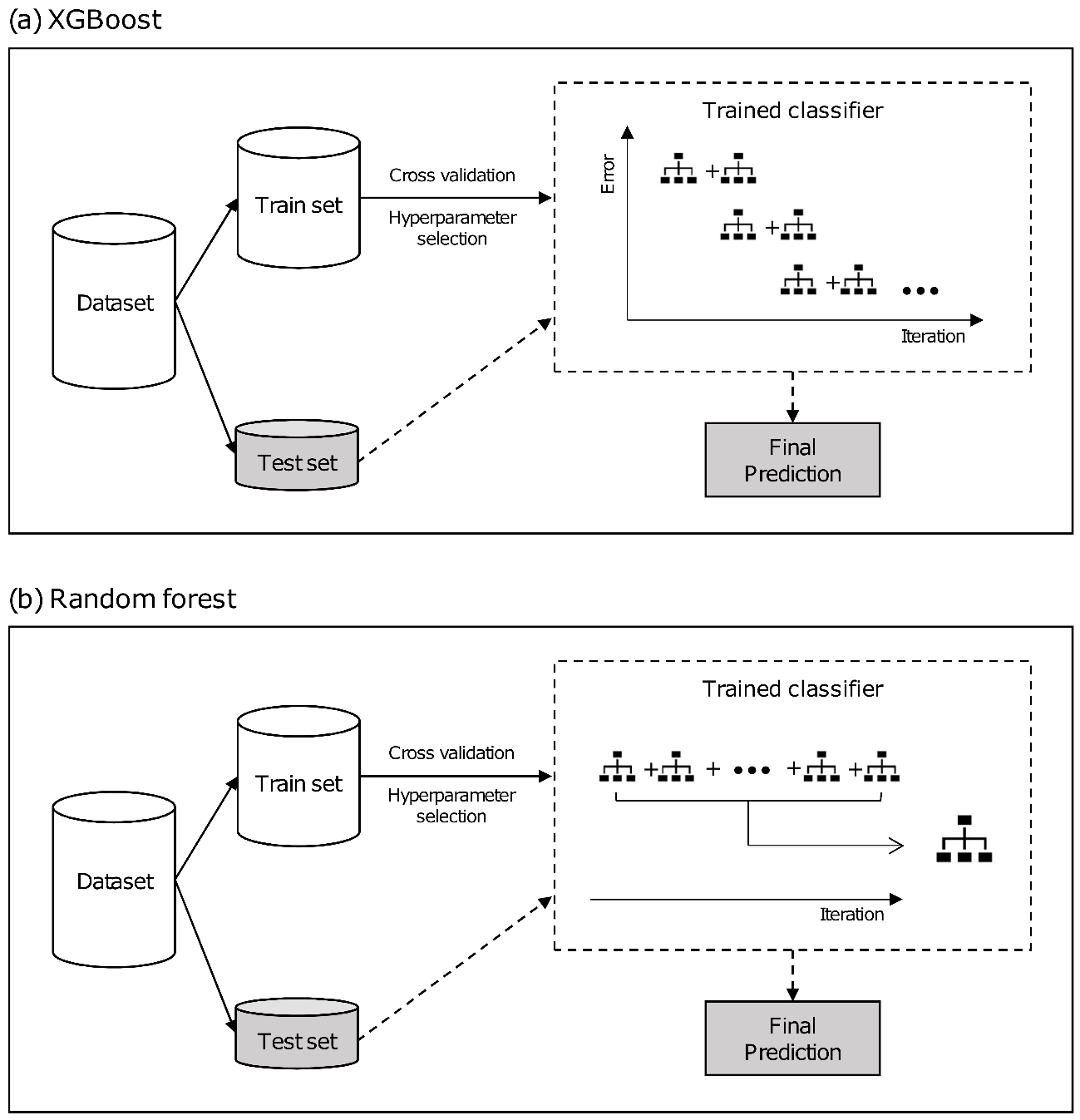
**

**Supplementary Fig. S3 Allelic diversity of 17 Y-STR markers across three geographical groups**


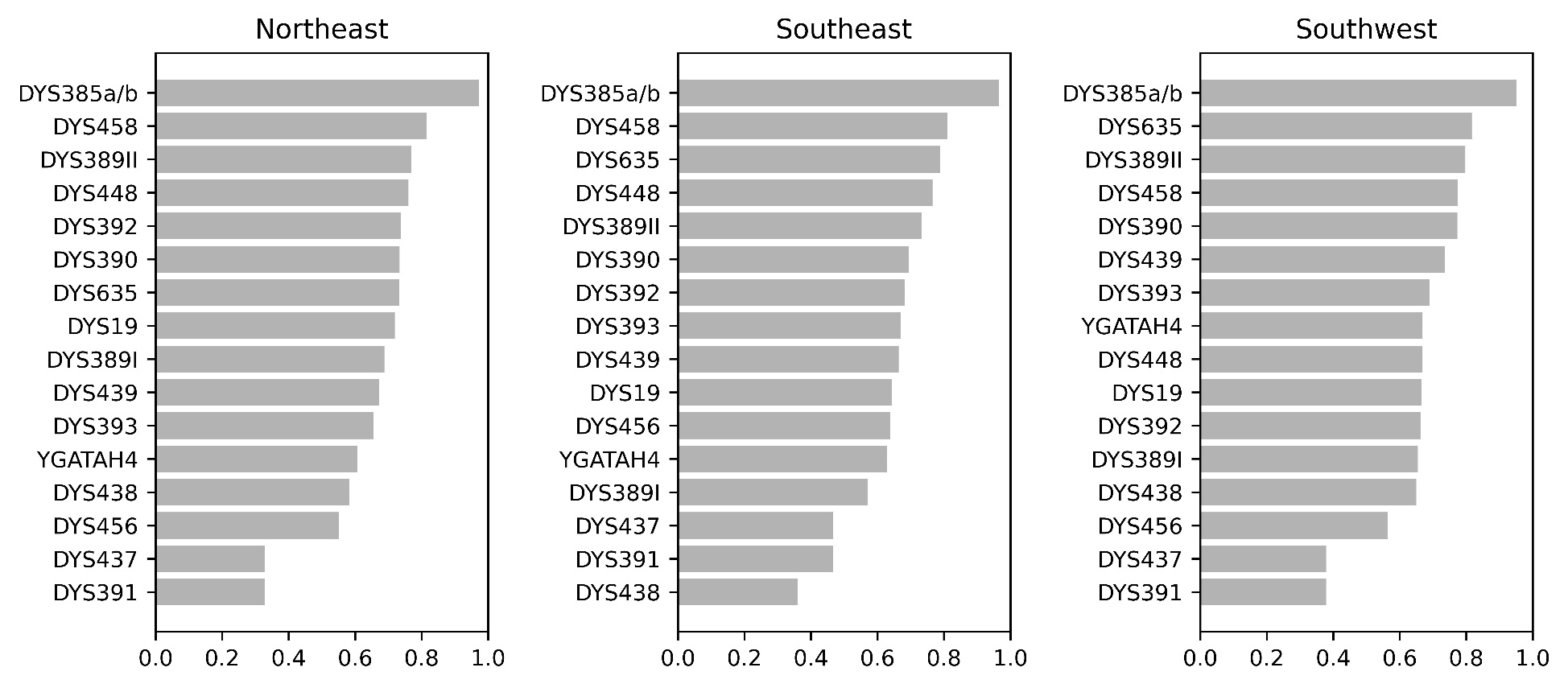


**Supplementary Fig. S4 Multidimensional scaling (MDS) plot illustrating the genetic relationships among 15 subgroups**

**
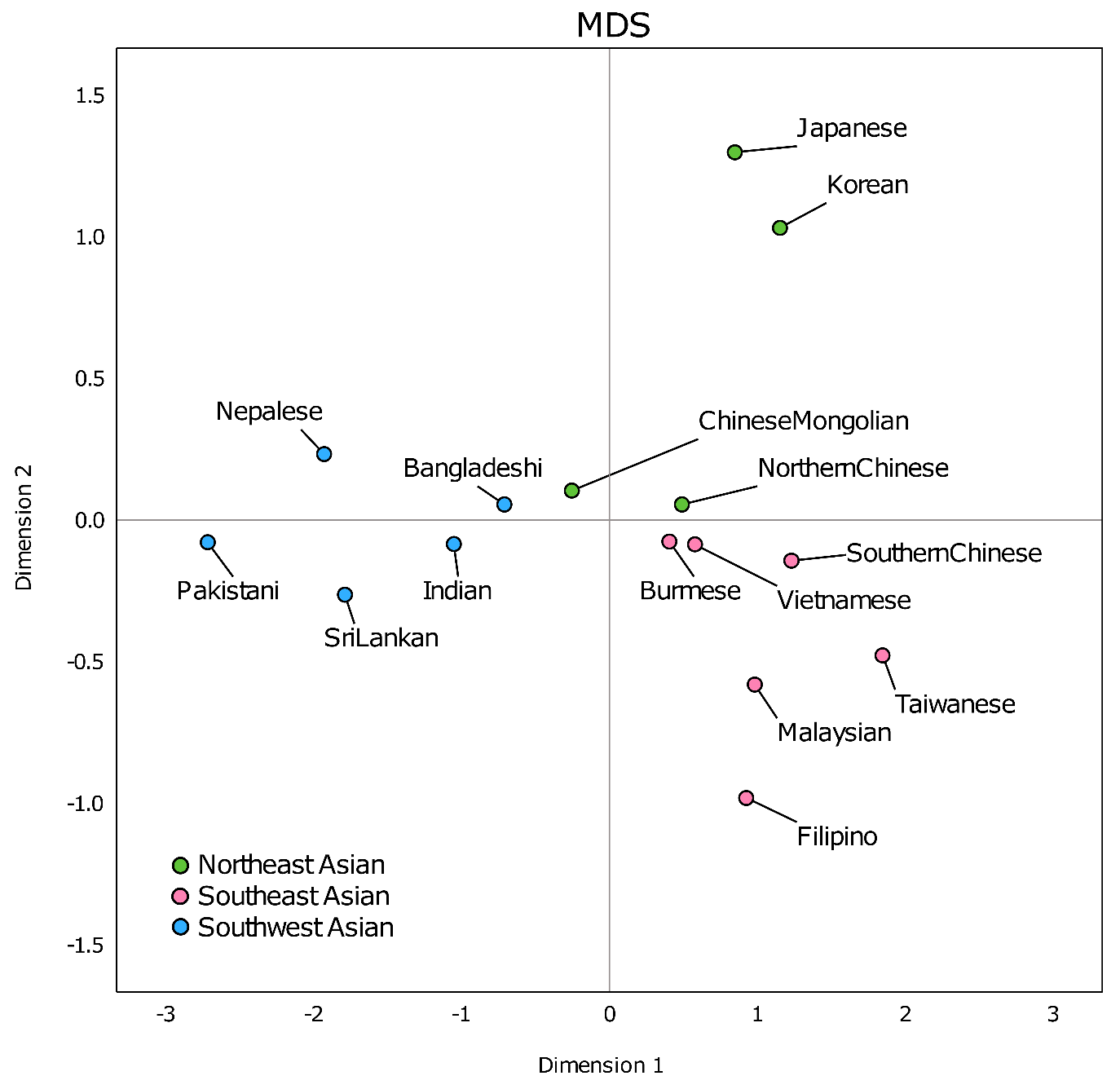
**

**Supplementary Fig. S5 Neighbor-joining (NJ) trees illustrating the genetic relationships among Asian populations**

(a) Three main geographical groups, and (b) Fifteen subgroups within these geographical groups

*Branch lengths shorter than 0.002 are omitted for clarity.

**
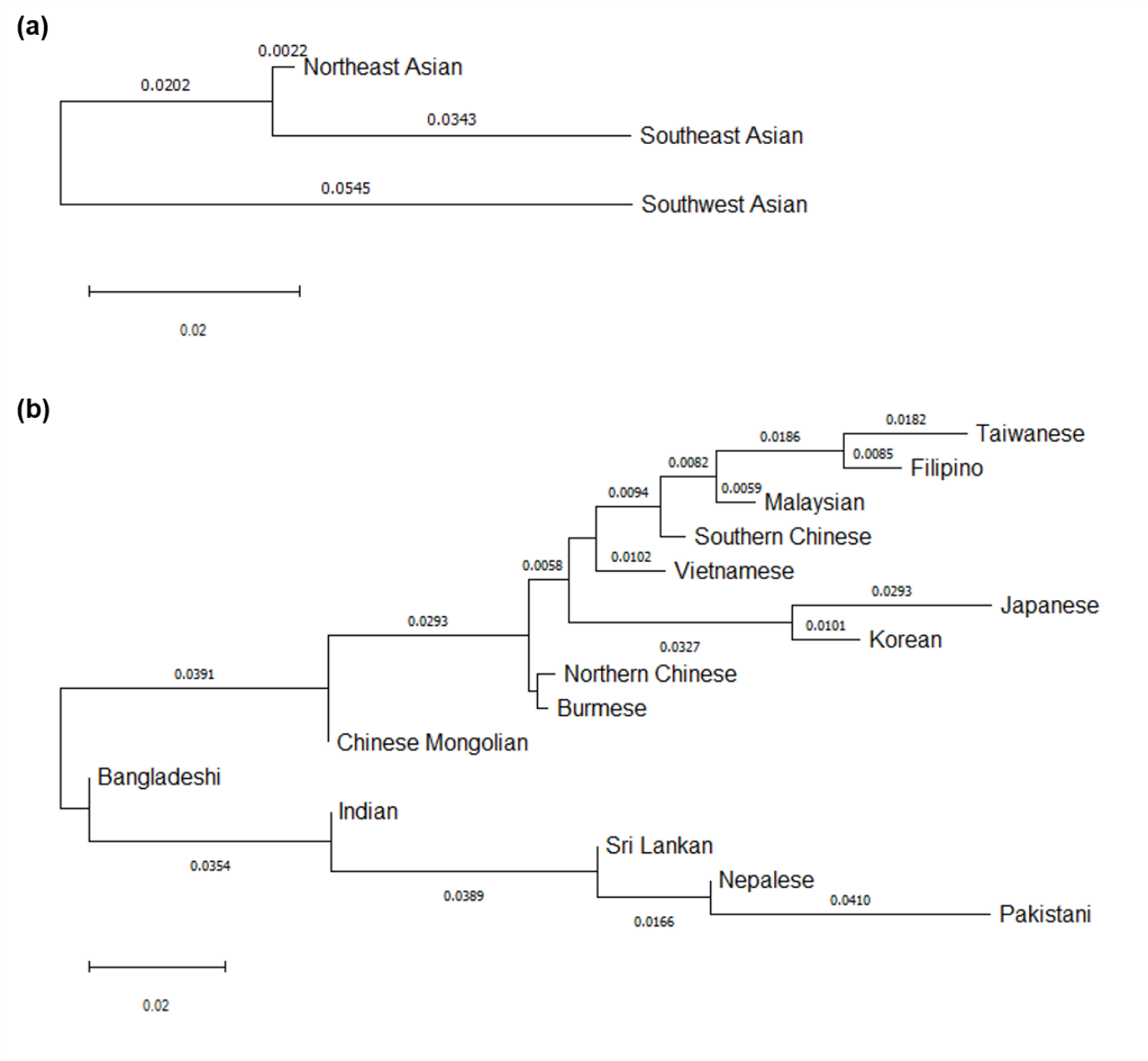
**
